# Empirical Law for the Magnetorheological Effect of Nanocomposite Hydrogels with Magnetite Microparticles

**DOI:** 10.3390/gels9030182

**Published:** 2023-02-25

**Authors:** Lukas Selzer, Stefan Odenbach

**Affiliations:** Institute of Mechatronic Engineering, Chair of Magnetofluiddynamics, Measuring and Automation Technology, Technische Universität Dresden, George-Bähr-Str. 3, 01062 Dresden, Germany

**Keywords:** nanocomposite hydrogel, NIPAAm, laponite, microparticles, magnetite, magnetorheological effect, rheology

## Abstract

Hydrogels are functional smart materials which can be tailored by modifying their chemical composition. Further functionalization can be achieved by incorporating magnetic particles into the gel matrix. In this study, a hydrogel with magnetite micro-particles is synthesized and characterized by rheological measurements. Inorganic clay is used as the crosslinking agent, which additionally prevents the sedimentation of the micro-particles during the synthesis of the gel. The mass fractions for the magnetite particles in the synthesized gels range from 10% to 60% in the initial state. Rheological measurements are performed in different degrees of swelling using temperature as a stimulus. The influence of a homogeneous magnetic field is analyzed by a step-wise activation and deactivation during dynamic mechanical analysis. For the evaluation of the magnetorheological effect in the steady states a procedure is developed, which takes occurring drift effects into account. Using the magnetic flux density, the particle volume fraction and the storage modulus as independent parameters, a general product approach is deployed for a regression analysis of the dataset. In the end, an empirical law for the magnetorheological effect in nanocomposite hydrogels can be found.

## 1. Introduction

By definition, hydrogels are hydrophilic porous networks with water as their swelling agent [1]. They are of scientific interest due to their good bio-compatibility and their application in sensors and actuators [2,3]. For many hydrogels, the amount of water absorbed by the network is dependent on external stimuli, which leads to a change of their volume and their mechanical properties. This stimuli-responsive swelling behavior can be tailored by modifying the molecular composition of the polymer chains [4,5]. The applied stimuli can range from temperature, pH, and salt concentration to electrical currents [6].

For further functionalization, magnetic particles can be incorporated into the polymeric network to create magnetoactive materials. In such magnetic composite materials, the mechanical properties can change depending on an external magnetic field. This effect is called the magnetorheological effect (MRE) [7]. Since the incorporation of nanoparticles in hydrogels by addition of the particles during the synthesis or by precipitation of particles inside a synthesized gel is fairly easy, such systems were studied at first in the past and were labeled as ferrogels [8,9]. In contrast, microparticles are rarely used, since sedimentation processes of such large particles complicate the synthesis of homogeneous samples.

In general, hydrogels can be synthesized by radical polymerization of a monomer with a crosslinking agent. A well known monomer for the synthesis of hydrogels is *N*-isopropylacrylamide (NIPAAm), which yields temperature sensitive hydrogels with a lower critical solution temperature (LCST) of 32 ${}^{\circ }C$ [10]. For organic hydrogels, *N*,*N*’-methylenbisacrylamide can be used as a crosslinking agent [11]. However, these hydrogels suffer from poor mechanical stability [12]. By using inorganic clay particles, such as Laponite, as the crosslinking agent, nanocomposite hydrogels can be synthesized, which are transparent and have good mechanical properties, which are the result of the surface interaction of the clay particles and the polymer chains after the synthesis [13]. A higher clay content correlates with a higher Young’s modulus [13]. Additionally, Laponite can influence the rheological properties of a solution and can form physical hydrogels due to electrostatic interaction of the clay particles by itself [14]. When using pure Laponite, this can limit the clay content, since the resulting reaction mixtures can be hard to handle. By adding sodium pyrophosphate, the rheological properties of the reaction mixture can be adjusted, to the point where a sol instead of gel is formed, even at high clay content [15].

In this study, we present a temperature sensitive nanocomposite hydrogel with magnetite micro-particles. We use NIPAAm as the monomer and Laponite as the crosslinking agent, which additionally prevents the sedimentation of the microparticles during the synthesis. We analyze the MRE of the material and the impact of the degree of swelling on the MRE. For this, we performed magnetorheological measurements with increasingly higher magnetic flux density for hydrogels with different magnetic particle content in the swollen state at $20\;{}^{\circ }C$ and the deswollen state at $40\;{}^{\circ }C$. To investigate the scaling behavior of the MRE, a regression analysis was performed using a general product approach. The magnetic flux density, the particle volume fraction and the storage modulus were selected as independent parameters. This yielded an empirical law for the MRE for this specific gel system.

## 2. Materials and Methods

### 2.1. Materials

Magnetite particles 48,806 (98.7%, $\rho =5.17\;g\;{\mathrm{cm}}^{-3}$) were purchased by Kremer Pigmente and sieved before usage. For deionized water, an ultrapure water filter PRO VE 3+ by AFT was used. Tetramethylethylenediamine (99%) and sodium persulfate (≥98.0%) were purchased from Sigma–Aldrich and *N*-isopropylacrylamide (stabilized by Mequinol, ≥98.0%) was purchased from Tokyo Chemical Industry. Laponite RD and Laponite RDS were obtained by BYK. All chemicals are used without further purification.

### 2.2. Sieving of Particles

Magnetite particles were dry sieved using a sieving tower by Haver and Boecker. Sieves with mesh sizes of 25 μm, 50 μm, 100 μm, and 200 μm were stacked and an amplitude of 3 mm was used for 15 min. The fraction from 25 μm to 50 μm of 200 g magnetite powder was collected and used for the synthesis.

### 2.3. Synthesis of Magnetorheological Nanocomposite Hydrogels

**Figure 1:**
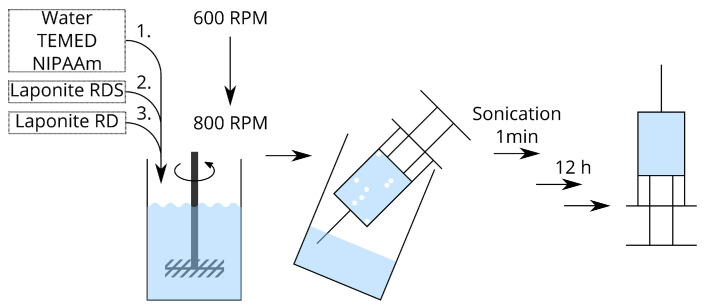
Procedure to prepare the pregel solution.

Deionized water (27 mL) is put into a 50 mL beaker and stirred at $600\;\min^{-1}$. Tetramethylethylenediamine (72 μL) and *N*-isopropylacrylamide (3 g) are added. After this is dissolved, Laponit RDS (1 g) is added. After this is dissolved, Laponit RD (2 g) is added slowly and the solution is stirred at $800\;\min^{-1}$ until everything is dissolved. The solution is then picked up into two 20 mL syringes and sonicated in a Elmasonic P 60 H by Elma for 1 min at 37 kHz to gather and remove air bubbles. This pregel-solution is stored for 12 h at room temperature before use. The preparation of the pregel-solution is shown in Figure 1.

Deionized water (3 mL) is put into a 5 mL Eppendorf tube. Sodium persulfate (94.6 mg) is added and mixed by inversion. This initiator solution is prepared directly before use.

**Figure 2:**
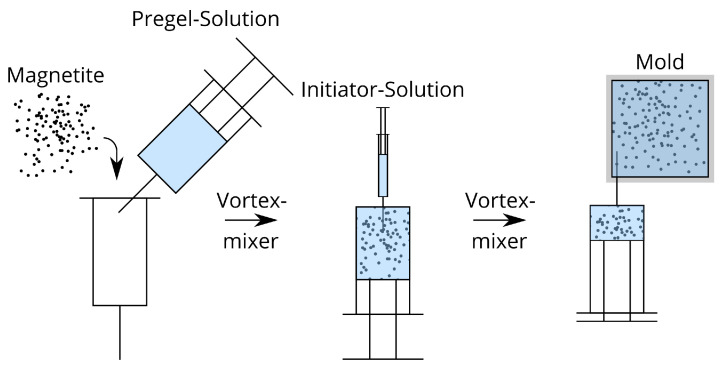
Procedure to prepare a magnetorheological hydrogel.

For the synthesis of a hydrogel the pregel-solution (5 g) is added to a 10 mL syringe. Magnetite particles are added according to Table 1. Using a vortex mixer the suspension is homogenized. Afterward, the initatior solution (0.45 g) is added into the syringe. The suspension is again homogenized using a vortex mixer and then injected into a mold with inner dimensions of 45 mm×45 mm×2 mm. After 24 h at room temperature, the polymerization reaction is completed. The preparation of a hydrogel is shown in Figure 2.

In total, two batches were synthesized. The first batch was synthesized with mass fractions from $w_{s}=10\%$ to $w_{s}=60\%$ in 10% intervals. For the second batch, only gels with $w_{s}=10\%$, $w_{s}=30\%$, and $w_{s}=60\%$ were synthesized for validation purposes.

### 2.4. Storage of Gels

The gels were stored in 200 mL deionized water within waterproof plastic vessels. These were placed into a water-bath WNB14 by Memmert for 14 days at $20\;{}^{\circ }C$. During this time, the storage solution was refreshed daily. After 14 days, rheological measurements were carried out at $20\;{}^{\circ }C$ by punching samples out of the hydrogels. After that, the storage temperature of the hydrogels was increased to $40\;{}^{\circ }C$ and the hydrogels were kept at that temperature for 7 days without refreshing the storage solution. Measurements at $40\;{}^{\circ }C$ were carried out thereafter by punching samples out of the hydrogels.

### 2.5. Determination of Degree of Swelling

Pictures of the gels were taken by a Xperia XZ2 by Sony. A 13 mm hole in a cardboard support was used as a reference. The four side lengths of the gels were determined manually using inkscape and averaged. The degree of swelling was calculated using $Q_{V}=V/V_{\mathrm{Ref}}$ with the initial state of synthesis as the reference volume $V_{\mathrm{Ref}}$ and the assumption of isotropic swelling.

### 2.6. Rheological Setup

Measurements were carried out using a Haake Mars III by ThermoFisher Scientific. A titanium plate was used as the lower measurement geometry while a 13 mm aluminum plate was used as the upper measurement geometry. The rheometer was modified with a custom magnetic setup, which is described in the thesis of J. Nowak [16]. Briefly, a copper coil at the bottom of the rheometer is used to generate a magnetic field, while an iron yoke is used to homogenize the magnetic field at the sample location. Water-channels in the lower section, as well as copper plates with water-channels inside the iron yoke are used with a thermostat for temperature control. The temperature was measured below the sample at the lower measurement geometry using a NTC-sensor. The readout was executed by an USB-6212 by National Instruments via a Python script. The temperature was regulated using a Viscotherm VT 2 by Anton-Paar. The magnetic field was generated using a GEN300-8 power supply unit by TDK-Lambda, which was controlled by a Python script. The magnetic flux density at the sample location was measured without a sample using a 5180 gaussmeter by F.W. Bell for calibration.

### 2.7. Rheological Measurements

Samples of 13 mm diameter are punched out of the gels using a STAS.01 sample punch by Q-tec. The samples are swabbed by a laboratory wipe to remove excess water from the surfaces of the samples. A small film of superglue is applied to the upper and lower measurement geometry. The sample is affixed to the upper geometry and then lowered by the rheometer until contact. Afterward, the normal force is set to 0.5 N. The sample is surrounded by 1 mL of storage solution to prevent drifts by evaporation. Dynamic mechanical analysis is used for all measurements. For all samples, amplitude- and frequency tests are performed to check for correct positioning and fixture. Magnetorheological measurements are carried out for 225 s at f=1 Hz and γ=1  in the swollen and γ=0.1% in the deswollen state. The magnetic field is activated at 45 s and deactivated at 135 s. Series of measurements are carried out this way for each sample using B=0 mT to $B=B_{\mathrm{Max}}$ in succession.

## 3. Results and Discussion

### 3.1. Magnetorheological Nanocomposite Hydrogels

Using nanocomposite hydrogels has two advantages. First, we obtain mechanically robust gels which are easier to handle for rheological measurements. Secondly, by using a mixture of Lapnoite RD and Laponite RDS, which contains sodium pyrophosphate, we achieved high clay contents, while preventing sedimentation of the magnetic micro-particles. However, the high viscosity of the reaction mixture required the use of syringes as vessels and did not allow the exclusion of air during the synthesis. Higher initiator concentrations were used for compensation.

### 3.2. Magnetorheological Measurements

#### 3.2.1. Measurement Conditions

Rheological measurements of gels can be challenging. Because of their constitution, wall slip effects must be considered if hydrogels are not synthesized within the measurement geometry. [17] By using superglue and a small normal force, those effects are prevented. Additionally, drift effects of various origins can occur. The gels have to be in a steady state for the measurement and are, thus, stored until a swelling equilibrium is reached. However, when placed inside the rheometer, the exact boundary conditions of the gel change. For example, small temperature gradients may occur since the cylindrical sample is in contact with the measurement geometry at the top and bottom side of the sample, while the atmosphere surrounds the mantle of the sample. Evaporation was detected to be a major contributor to drift effects and was prevented by surrounding the sample with the storage solution. This minimized the drift effects. While in theory, measurements could be conducted for each sample after a new equilibrium within the rheometer was reached, it was observed that drift effects had random initial directions and could change direction even hours after placing the sample in the setup. Instead, a method for the evaluation of the MRE with consideration of drift effects was developed.

#### 3.2.2. Evaluation Method

The MRE is defined to be the relative increase in a given module under the influence of a magnetic field:

(1) $$\mathrm{MRE}=\frac{G(B)}{G_{0}}-1$$

The drift is assumed to be a constant linear increase $\frac{\Delta G}{\Delta t}$ of the modulus, which is independent of the MRE. In our experiments, the magnetic field is activated at $t_{A}$ and deactivated at $t_{D}$, thus:

(2) $$G\left(t\right)=\left\{\begin{array}{cc} \left(G_{0}+\frac{\Delta G}{\Delta t}\;\cdot \;t\right) & t<t_{A}\\ \left(G_{0}+\frac{\Delta G}{\Delta t}\;\cdot \;t\right)\;\cdot \;\left(1+\mathrm{MRE}\right) & t_{A}<t<t_{D}\\ \left(G_{0}+\frac{\Delta G}{\Delta t}\;\cdot \;t\right) & t>t_{D} \end{array}\right.$$

**Figure 3:**
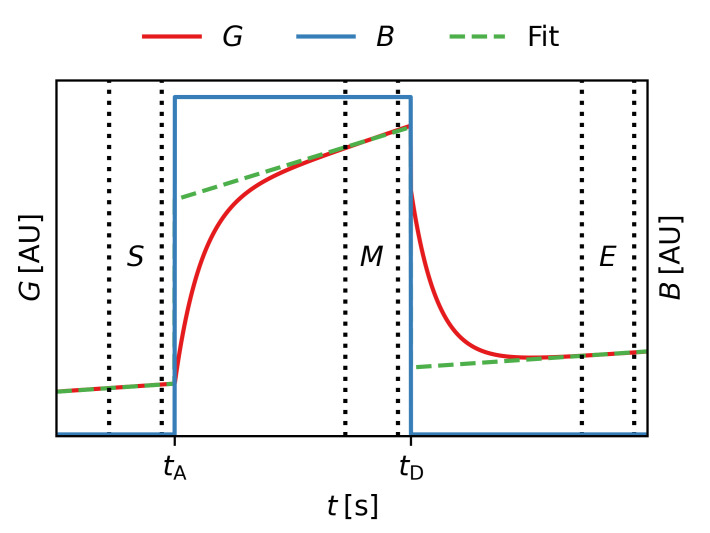
Evaluation of magnetorheological effect under consideration of drift effects using Equation (2) with idealized data. Only data points in the intervals S, M, und E are used for regression analysis.

In order to analyze the steady states, only data points in intervals before the activation of the magnetic field, before the deactivation of the magnetic field and at the end of the measurement are used for a fitting procedure. The idealized process is shown in Figure 3.

**Figure 4:**
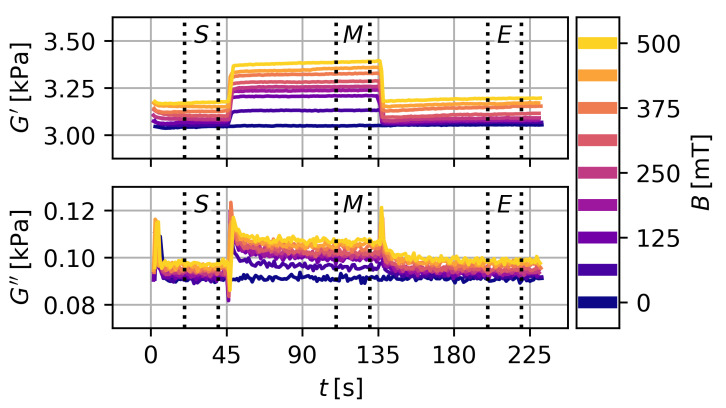
Storage and loss modulus over time. The magnetic field is activated at t=45 s and deactivated at t=135 s. Measurements are conducted successively with increasing magnetic flux density for a single sample. $w_{s}=10\;$ and $T=20\;{}^{\circ }C$.

With this method the value of the modulus, the occurring drift and the magnetorheological effect can be determined. Exemplary data for $T=20\;{}^{\circ }C$ and $w_{s}=10\%$ is shown in Figure 4. The storage modulus shows a stepwise increase and decrease with the activation and deactivation of the magnetic field. The loss modulus shows an overshoot at the activation with a subsequent relaxation to an increased level and an overshoot at the deactivation with a subsequent relaxation back to the base level. Data for higher particle content or at $T=40\;{}^{\circ }C$ show qualitatively the same behavior. However, drift effects are more pronounced at $T=40\;{}^{\circ }C$. Additional data are shown in the Supplementary Information.

### 3.3. Nonmagnetic Influences on the Mechanical Properties

**Figure 5:**
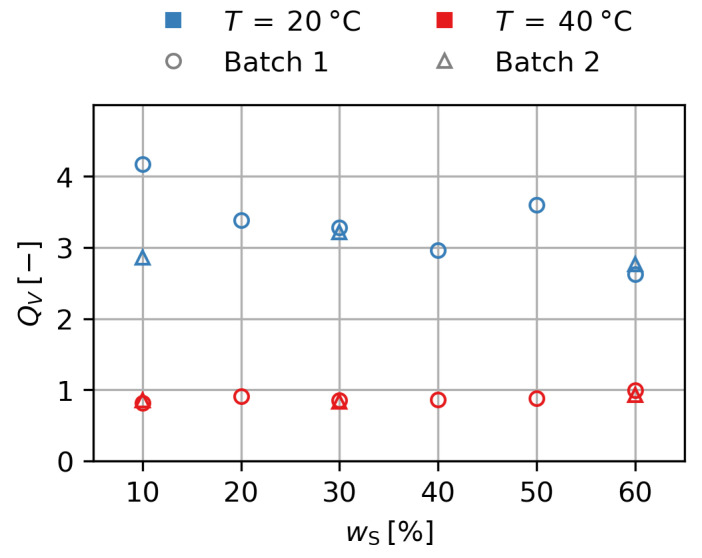
Degree of swelling over mass fraction of particles in the initial state for the first and second batch of gels.

In order to understand the magnetic behavior, first the nonmagnetic behavior of the materials has to be analyzed. Figure 5 shows the degree of swelling $Q_{V}$ over the mass fraction of particles in the initial state $w_{s}$. All gels of batches 1 and 2 show a degree of swelling of $Q_{V}=3.2\pm 0.9$ at $T=20\;{}^{\circ }C$ and of $Q_{V}=0.9\pm 0.1$ at $T=40\;{}^{\circ }C$. While there is a higher variance for $T=20\;{}^{\circ }C$, there is no significant influence of $w_{s}$ for $Q_{V}$. The volume increase by swelling leads to a decrease in the particle content, since $\phi_{p}=\phi_{p,0\;}/Q_{V}$. While $w_{s}$ can be used as an identifier for single gels, any scaling of effects will be influenced by the actual particle content $\phi_{p}$, which will be used for further considerations.

**Figure 6:**
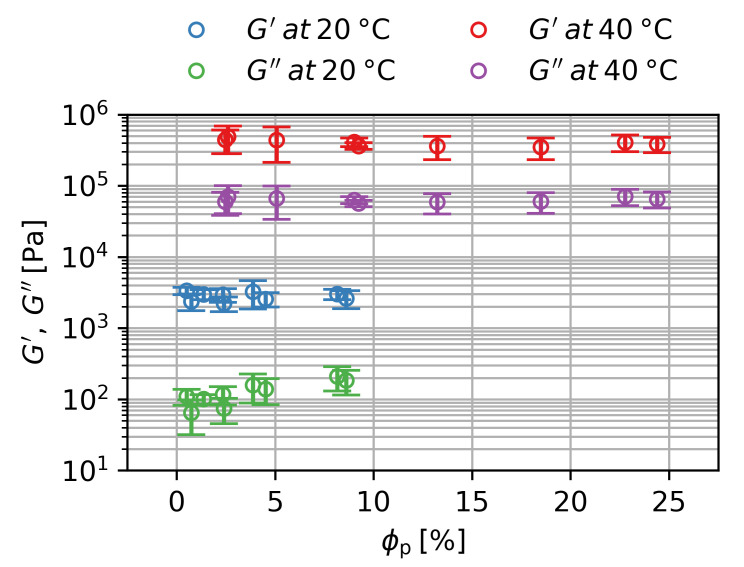
Storage and loss modulus over volume fraction of particles for the first and second batches. Averaged values for three samples per $\phi_{p}$ with two standard deviations as statistical error are shown.

Figure 6 shows the storage modulus $G^{\prime }$ and the loss modulus $G^{\prime \prime }$ over the volume fraction of the particles $\phi_{p}$ for the swollen and deswollen state for the first and second batch. The storage modulus increases from $G^{\prime }=3\pm 1\;k\mathrm{Pa}$ in the swollen state to $G^{\prime }=400\pm 150\;k\mathrm{Pa}$ in the deswollen state by a factor of 130, while the loss modulus increases from $G^{\prime \prime }=0.13\pm 0.12\;k\mathrm{Pa}$ in the swollen state to $G^{\prime \prime }=60\pm 20\;k\mathrm{Pa}$ in the deswollen state by a factor of 460. An increase in the moduli by deswelling is in general expected and according to the Flory–Rehner theory, which describes the general swelling behavior of polymeric networks [18]. However, the observed changes are about two orders of magnitude higher than expected by the Flory–Rehner theory. This can be explained by the composition of nanocomposite hydrogels, which is very different compared to what is assumed in the Flory–Rehner theory. Long polymer chains interact with multiple clay particles, building up the network [19]. Due to the ionic nature of clay particles, the gel also has to be considered to be a polyelectrolyte network. Additionally, a coil-to-globule-transition occurs at the deswelling process, which changes the chain distribution and is not considered in the Flory–Rehner theory [20]. All these factors lead to a bigger increase in the moduli when the gels are deswelling.

An increase in $\phi_{p}$ leads to a significant increase in $G^{\prime \prime }$ at $T=20\;{}^{\circ }C$. There is no significant influence of $\phi_{p}$ for $G^{\prime \prime }$ at $T=40\;{}^{\circ }C$ and no influence of $\phi_{p}$ for $G^{\prime }$ at either temperature. Therefore, particles do not seem to contribute to the mechanical integrity of the hydrogel, but only increase the friction in the swollen state when the sample is sheared. Since there is a higher baseline friction in the deswollen state at $T=40\;{}^{\circ }C$, the contribution of the particles to the loss modulus is negligible. The magnetite particles also do not seem to have any influence on the polymerization reaction, as this would also impact the mechanical properties.

### 3.4. Regression Analysis for the Magnetorheological Effect

**Figure 7:**
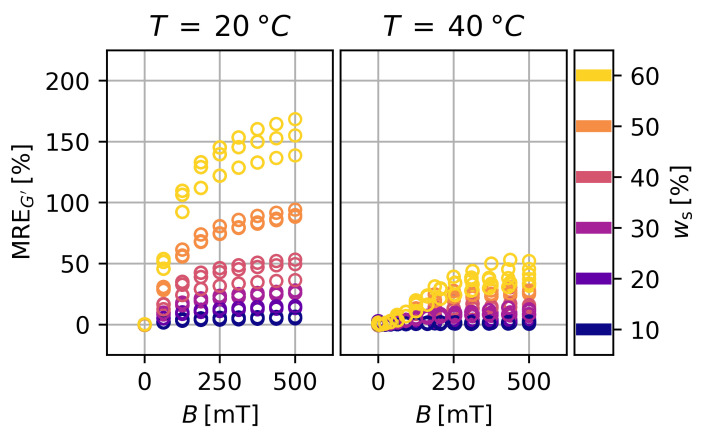
Magnetorheological effect of the storage modulus over the magnetic flux density for gels with different mass fractions of particles in the initial state for the first batch of gels. Three samples per $w_{s}$ are shown. Error bars are smaller than the symbols.

Figure 7 shows the magnetorheological effect of the storage modulus ${\mathrm{MRE}}_{G^{\prime }}$ over the magnetic flux density *B* for gels with different mass fractions of particles in the initial state $w_{s}$ at $T=20\;{}^{\circ }C$ and $T=40\;{}^{\circ }C$. Roughly summarized, the MRE increases with the magnetic flux density and reaches a saturation at high-flux densities. Additionally, the MRE increases with the particle content and is diminished for the deswollen state. The MRE of the loss modulus behaves qualitatively the same. It has to be noted that the MRE is small compared to the changes exhibited by the gels by deswelling. The maximum MRE observed increases the module by a factor of 2.5, while factors over 100 can be observed for deswelling.

For a regression analysis, a general product approach is used with the magnetic flux density, the particle volume fraction and the storage modulus as independent parameters. Thus:

(3) $$\mathrm{MRE}\left(B,\phi_{p},G^{\prime }\right)=K_{p}\;\cdot \;f\left(B\right)\;\cdot \;g\left(\phi_{p}\right)\;\cdot \;h\left(G^{\prime }\right)$$

$K_{p}$ denotes a material constant which implies the saturation magnetization of the particles, as well as an interaction constant between the particles and the matrix material. The functions *f*, *g*, and *h* represent the yet unknown relationship between the MRE and the parameters. It is assumed that there is no interaction between the parameters and, thus, no cross terms. $G^{\prime }$ was chosen as the influence of the mechanical properties of the matrix on the MRE, since $G^{\prime }$ describes the elastic properties of the material which can have an impact on steady states, while $G^{\prime \prime }$ can only have an impact on the dynamic behavior.

In order to determine *f*, the dataset of each sample is normalized to the MRE at $B=B_{\max}$. Since all other parameters are constant, only the normalized function $f^{*}$ remains:

(4) $$f^{*}\left(B\right)=\frac{f\left(B\right)}{f\left(B=500\;mT\right)}=\frac{\mathrm{MRE}\left(B,\phi_{p},G^{\prime }\right)}{\mathrm{MRE}\left(B=500\;mT,\phi_{p},G^{\prime }\right)}$$

**Figure 8:**
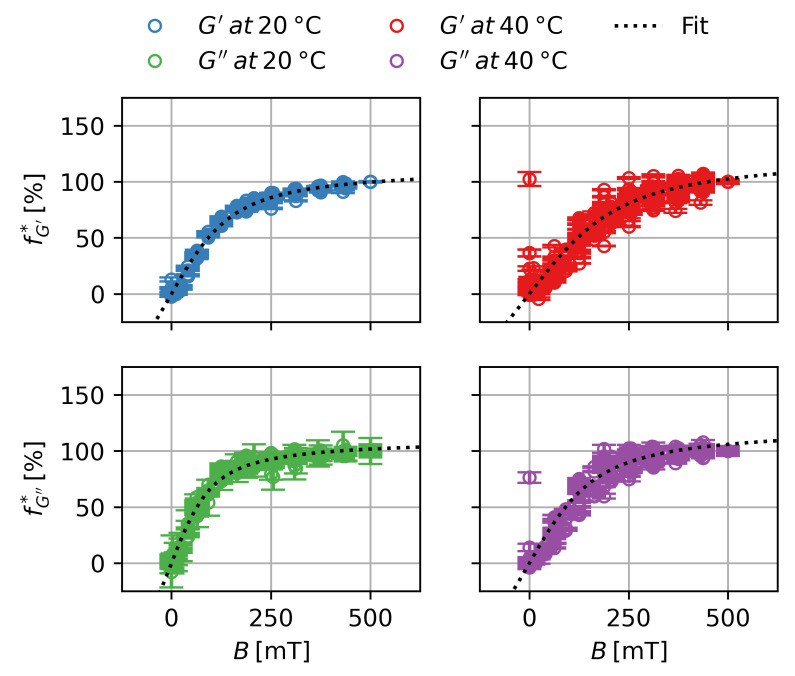
Magnetorheological effect normalized to the maximum effect at B=500 mT over the magnetic flux density for the storage and loss modulus in the swollen and deswollen state.

This is shown in Figure 8. Each subplot shows the normalized datasets of 27 different samples ranging from $w_{s}=10\%$ to $w_{s}=60\%$ of two batches of gels. In the case of $G^{\prime }$ at $T=20\;{}^{\circ }C$ an almost perfect universal scaling behavior can be observed. For $G^{\prime \prime }$ a small increase in variance can be detected, while major variations occur for $T=40\;{}^{\circ }C$ for $G^{\prime }$ and $G^{\prime \prime }$.

The behavior can be well approximated by a modified Langevin-function. $K^{*}$ and $B^{*}$ are fit-parameters to enable vertical and horizontal stretching of the function. Fits were carried out for each modulus at $T=20\;{}^{\circ }C$ and $T=40\;{}^{\circ }C$ separately. The parameters are shown in Table 2.

(5) $$f^{*}\left(B\right)=K^{*}\;\cdot \;L\left(\frac{B}{B^{*}}\right)=K^{*}\;\cdot \;\left(\coth\left(\frac{B}{B^{*}}\right)-\frac{B^{*}}{B}\right)$$

The universal scaling of the MRE for $G^{\prime }$ and $G^{\prime \prime }$ at $T=20\;{}^{\circ }C$ shows that there is no influence of the particle content $\phi_{p}$ on the normalized MRE and, thus, no influence on the scaling with the magnetic field. The higher relative variance in the case of $G^{\prime \prime }$ is due the lower absolute values of the measurements of $G^{\prime \prime }$. There is a significant difference between the scaling of ${\mathrm{MRE}}_{G^{\prime }}$ and ${\mathrm{MRE}}_{G^{\prime \prime }}$ with the magnetic field. Since they describe different phenomena—one being the increase in the storage modulus while the other being the increase in the loss modulus—the exact same scaling behavior can not be expected. The similarities point to a scaling with the magnetization of the sample with different coefficients. For the deswollen state at $T=40\;{}^{\circ }C$ a higher variance for the MRE can be observed which can be linked to the lower values of the MRE and more dominant drift effects. For ${\mathrm{MRE}}_{G^{\prime \prime }}$ a significant increase in $B^{*}$ can be observed. This could either indicate an influence of $G^{\prime }$ or *T* on the scaling, or be an artifact of the higher variance and drift effects for those measurements. Nonetheless, as a generalization it can be concluded that the magnetic scaling can be well approximated by a Langevin-function and, thus:

(6) $$f\left(B\right)=L\left(B\right)$$

Since no significant influence of $\phi_{p}$ on $G^{\prime }$ was observed for our samples and saturation of the MRE is reached for high magnetic fields, the influence of $\phi_{p}$ on the MRE can be analyzed by using a reference state ${\mathrm{MRE}}_{\mathrm{Ref}}$ at either the swollen or deswollen state at B=500 mT. The influence of the magnetic field and the storage modulus results in a constant *K*, while the influence of the particles is represented by the function $g\left(\phi_{p}\right)$:

(7) $$\mathrm{MRE}\left(B=500\;mT,\phi_{p},G^{\prime }=\mathrm{const}.\right)={\mathrm{MRE}}_{\mathrm{Ref}}\left(\phi_{p}\right)=K\;\cdot \;g\left(\phi_{p}\right)$$

**Figure 9:**
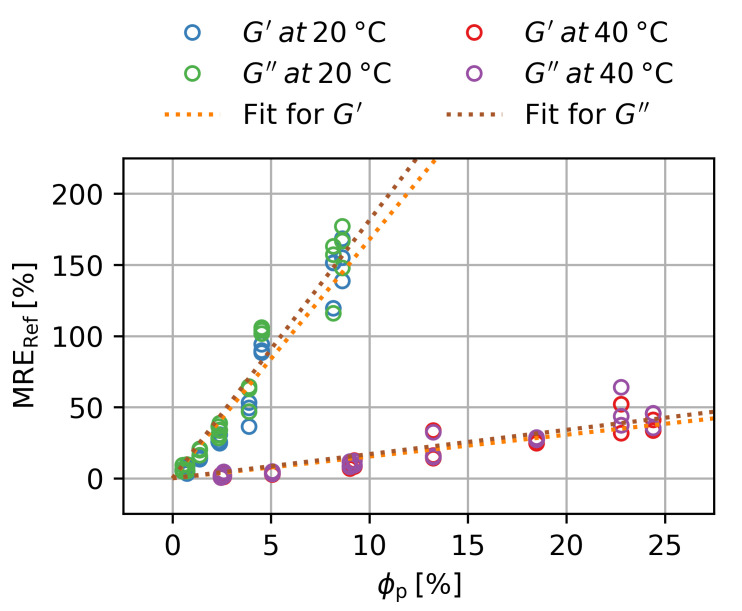
Magnetorheological effect at B=500 mT over the particle volume fraction for the storage and loss modulus in the swollen and deswollen state. Three samples per $w_{s}$ are shown. Error bars are smaller than the symbols.

The reference states are shown in Figure 9. The MRE seems to be a directly proportional to $\phi_{p}$. The slope *K* for all cases was determined and is shown in Table 3. There are no significant differences between $K_{G^{\prime }}$ and $K_{G^{\prime \prime }}$, while the slopes decrease when the samples are in the deswollen state. This is caused by the increase in $G^{\prime }$ due to deswelling. As a generalization, we can record that:

(8) $$g\left(\phi_{p}\right)=\phi_{p}$$

Since the mathematical relationship between MRE and $\phi_{p}$ is now known, we can use the ${\mathrm{MRE}}_{\mathrm{Ref}}$ normalized by $\phi_{p}$ to analyze the pure influence of $G^{\prime }$ on the MRE:

(9) $$\frac{\mathrm{MRE}\left(B=500\;mT,\phi_{p},G^{\prime }\right)}{\phi_{p}}=\frac{{\mathrm{MRE}}_{\mathrm{Ref}}\left(\phi_{p},G^{\prime }\right)}{\phi_{p}}=K_{p}\;\cdot \;h\left(G^{\prime }\right)$$

**Figure 10:**
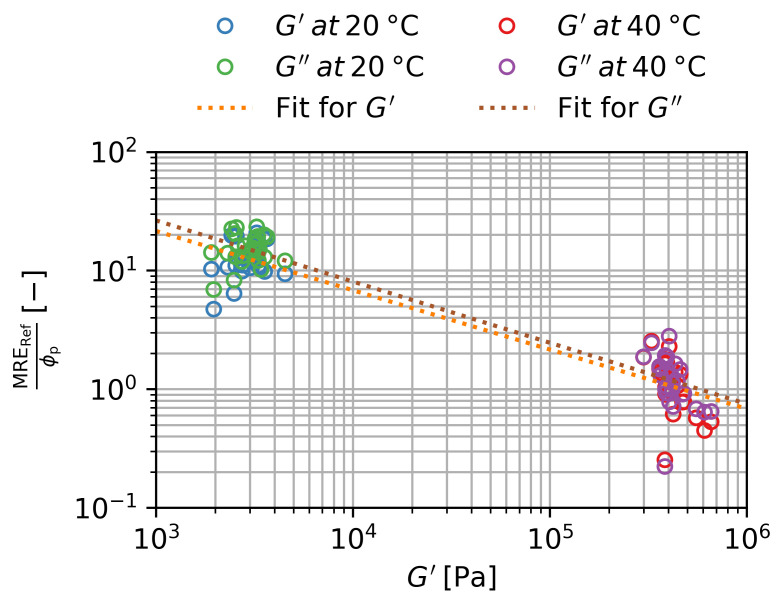
Magnetorheological effect at B=500 mT normalized to the particle volume fraction over the storage modulus for the storage and loss modulus in the swollen and deswollen state. Three samples per $w_{s}$ are shown. Error bars are smaller than the symbols.

This is shown in Figure 10 in double logarithmic scale. There is a clustering of the data for the swollen and the deswollen state. Assuming a simple logarithmic relationship, the constant *C* and the exponent *n* can be determined:

(10) $$\log\left(\frac{{\mathrm{MRE}}_{\mathrm{Ref}}\left(\phi_{p},G^{\prime }\right)}{\phi_{p}}\right)=n\;\cdot \;\log\left(G^{\prime }\right)+C$$

The parameters are shown in Table 4. Again there is no significant difference between the scaling of ${\mathrm{MRE}}_{G^{\prime }}$ and ${\mathrm{MRE}}_{G^{\prime \prime }}$ and in both cases an exponent of n≈−0.5 and a constant C≈6.5 can be determined. With this we can record that:

(11) $$h\left(G^{\prime }\right)=\frac{1}{\sqrt{G^{\prime }}}$$

Combining all previous observations, we can find that:

(12) $$\mathrm{MRE}\left(B,\phi_{p},G^{\prime }\right)=\frac{K_{p}\;\cdot \;L\left(B\right)\;\cdot \;\phi_{p}}{\sqrt{G^{\prime }}}$$

This is an empirical scaling law for the MRE of nano-composite hydrogel with micro-scaled magnetite particles. A scaling with $L\left(B\right)$ implies a direct scaling of the MRE with the magnetization of the sample. Considering the typical scaling behavior of magnetic forces, a scaling with $L\left(B\right)\;\cdot \;B$ was expected instead. The MRE of $G^{\prime }$ and $G^{\prime \prime }$ only differ in their exact scaling with *B* with an earlier onset of saturation in the case of $G^{\prime \prime }$. A scaling with $\phi_{p}$ implies that there is a scaling with the content of the magnetic material within a sample, but also that there is no interaction between the magnetic particles which contributes to the MRE. Interactions between particles typically scale with $r^{-n}$. This would result in a scaling of a higher order for $\phi_{p}$ than observed, since $r\propto \phi_{p}^{-\frac{1}{3}}$. An inverse scaling with $G^{\prime }$ can be explained by a movement or rotation of the particles when a magnetic field is active, which is hindered by the stiffness of the matrix material. It is unclear why precisely a scaling with −0.5 is observed. Additionally, because of the heavy clustering of the data points in effectively two points, this mathematical relationship can be considered to be the least reliable.

### 3.5. Degree of Swelling as a Parameter

For a single gel an approach using the degree of swelling as a parameter is more useful since for a synthesized gel the particle content $\phi_{p}$ and storage modulus $G^{\prime }$ are both dependent on $Q_{V}$. In the case of $\phi_{p}$ there is the relationship of $\phi_{p}=\phi_{p,0\;}/Q_{V}$, where $\phi_{p,0\;}$ is the particle concentration in the initial state of synthesis. In the case of $G^{\prime }$ a general power-law approach of $G^{\prime }\propto Q_{V}^{n}$ can be assumed. While the Flory–Rehner theory yields $n=-\frac{1}{3}$ for this approach, theories for polyelectrolyte gels at high salt concentration can yield values of n=−2  [21]. Using both relationships in Equation (12) we find:

(13) $$\mathrm{MRE}\left(B,Q_{V}\right)=\frac{K_{\mathrm{Gel}}\;\cdot \;\phi_{p,0\;}\;\cdot \;L\left(B\right)}{Q_{V}^{1+\frac{n}{2}}}$$

**Figure 11:**
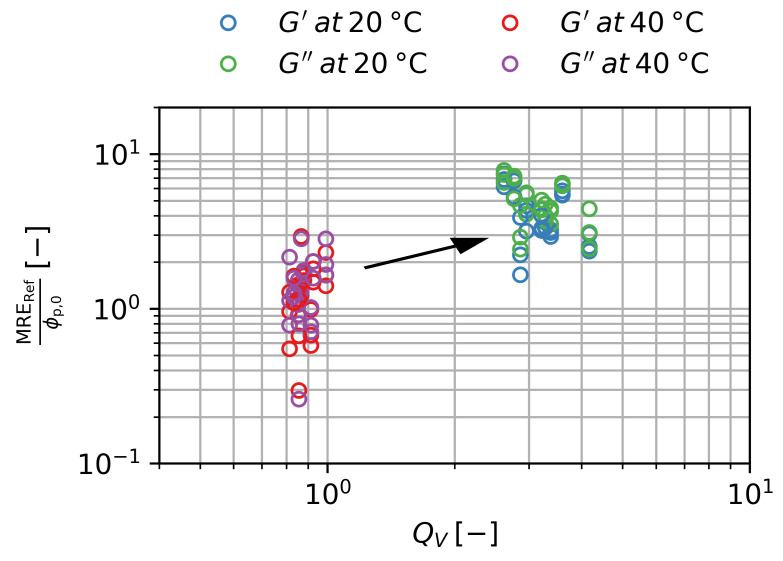
Magnetorheological effect at B=500 mT normalized to the initial particle volume fraction over degree of swelling for the storage and loss modulus. Three samples per $w_{s}$ are shown. Error bars are smaller than the symbols.

An increase in $Q_{V}$ has two different impacts on the MRE. On the one hand, an increase leads to a decrease in $\phi_{p}$ and, hence, decreases the MRE. On the other hand, $G^{\prime }$ decreases with $Q_{V}$ and thus increases the MRE. The final scaling of MRE with $Q_{V}$ is consequently dependent on the precise scaling exponent *n* of $G^{\prime }$ with $Q_{V}$. In the case of the Flory–Rehner theory, a net negative scaling of $\mathrm{MRE}\propto Q_{V}^{-\frac{5}{6}}$ would be expected, while the MRE is expected to be constant independently of $Q_{V}$ for n=−2  in the case of polyelectrolyte gels at high salt concentration. In our data, we find n<−2 , which leads to a positive scaling of the MRE with $Q_{V}$. This is shown in Figure 11 using ${\mathrm{MRE}}_{\mathrm{Ref}}$ normalized by $\phi_{p,0\;}$.

### 3.6. Statistical Analysis

To support our approach, we checked the statistical significance of our models. Since we used a piecewise function for the evaluation of the MRE, *p*-values are not well-defined. However, the approximation of $G^{\prime }$ or $G^{\prime \prime }$ with a linear function can be evaluated. For this, we used the statsmodel library of Python, using the data points before the activation of the magnetic field and at the end of the measurement, but not the data points before the deactivation of the magnetic field. As shown in Table 5, for all 648 measurement curves, we find significant *p*-values for the intercepts of all the regressions. Only for a few measurement curves, *p*-values of the slopes are insignificant. On closer inspections, these curves exhibit a change of the drift direction. Nevertheless, and even in these cases, the used procedure is a good approximation for $G^{\prime }$ and $G^{\prime \prime }$ and, subsequently, the evaluation of MRE.

**Figure 12:**
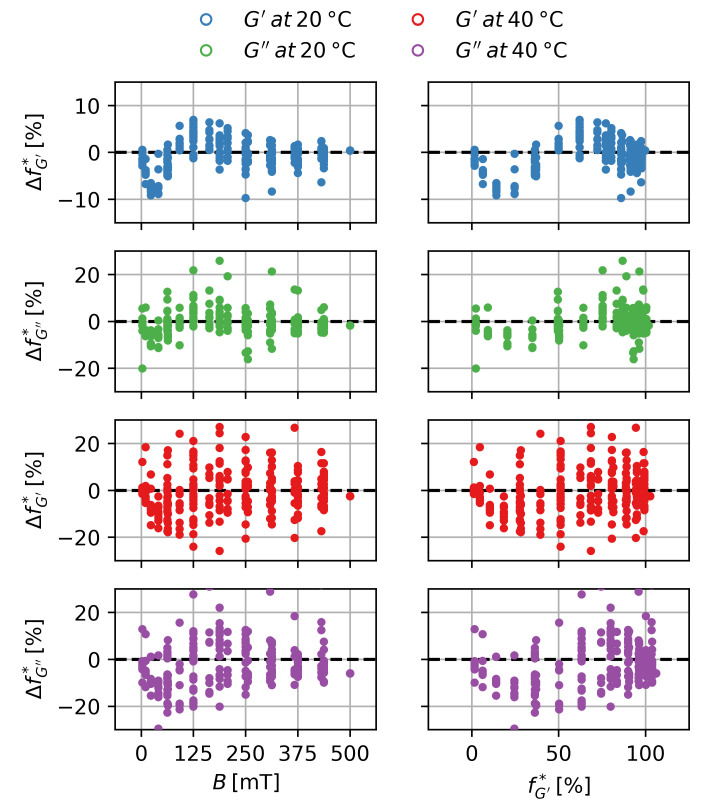
Residuals over the independent value *B* and the fitted value $f^{*}$.

As the Langevin-function is also a non-linear function, again *p*-values are not well-defined. In this case, we resort to a graphical evaluation of the residuals. These are shown in Figure 12 over the independent value *B* and the fitted value $f^{*}$. For $T=40\;{}^{\circ }C$, the residuals are distributed randomly in good approximation in the cases of $G^{\prime }$ and $G^{\prime \prime }$. For $T=20\;{}^{\circ }C$, especially in the case of $G^{\prime }$, a wave-like pattern does occur. It has to be noted that the residuals of this case are smaller than in the other cases. This pattern indicates that the Langevin-function fails to perfectly model the experimental data for very low magnetic flux densities. However, as shown in the other cases, the Langevin-function still appears to be a good approximation for the scaling behavior of the MRE with *B*. Additionally, it is a known mathematical function in physics describing the scaling behavior with the magnetic flux density, which supports the use as an approximation.

In the case of $g\left(\phi_{p}\right)$ we can resort to *p*-values and $R^{2}$-values, which are shown in Table 6. Both values indicate a good agreement between the experimental data and the regression, justifying to use a simple linear function for $g\left(\phi_{p}\right)$.

In the case of $h\left(G^{\prime }\right)$, by applying the logarithmic transformation to the data, *p*-values and $R^{2}$-values can be used. These are shown in Table 7. Both values indicate a good agreement between the experimental data and the regression, justifying to use a simple power law for $h\left(G^{\prime }\right)$.

## 4. Conclusions

In our study, we presented the synthesis of nanocomposite hydrogels with magnetite micro-particles. We avoided sedimentation by using clay particles as crosslinking agents and adjusting the rheological properties of the reaction mixture by sodium pyrophosphate. For magnetorheological measurements, we prevented wall-slip effects and minimized drift effects. As those drifts could not be eliminated completely, we developed a procedure to extract the mean mechanical properties and the value of the MRE under consideration of those drifts. For our hydrogel system, the degree of swelling has an extremely high impact on the mechanical properties of the hydrogels, which exceeds what can be expected by typical theories. Particles do not seem to contribute to the mechanical integrity of the gels in the absence of a magnetic field, and only increase the baseline friction in the swollen state of the gels. Using a regression analysis with a general product approach, we found an empirical law for the MRE with *B*, $\phi_{p}$ and $G^{\prime }$ as independent parameters. By analyzing the scaling behavior of the MRE, the underlying physical processes can be understood. Notably, the MRE has a smaller scaling behavior with the magnetic flux density than what would be expected by the typical scaling of magnetic forces. It is also surprising that particle interaction does not seem to contribute to the observed MRE, as for many magnetorheological systems, chain-formation of particles is a key mechanism for the MRE in those systems [22]. Additionally, the degree of swelling was considered as an independent parameter, which is unique to hydrogels. The degree of swelling impacts the particle concentration, as well as the stiffness of the material, and by extension impacts the MRE. The final scaling of the MRE with $Q_{V}$ is highly dependent on the scaling behavior of $Q_{V}$ and $G^{\prime }$, which can lead to a net negative, positive or even constant scaling behavior. This relationship must be considered for any application of magnetorheological hydrogels. In a later study, we will propose a theoretical mechanism for the underlying physical processes for this system supported by tomographic data.

## Figures and Tables

**Table 1:** Composition of synthesized magnetorheological gels. $w_{s}$ and $\phi_{p,0\;}$ denote the mass and volume fraction of the particles in the initial state of synthesis.

$w_{s}\;$ [%]	$\phi_{p,0\;}\;$ [%]	$m_{\mathrm{pregel}}\;\left[g\right]$	$m_{\mathrm{initiator}}\;\left[g\right]$	$m_{\mathrm{particles}}\;\left[g\right]$
10	2.1	5	0.45	0.61
20	4.6	5	0.45	1.36
30	7.7	5	0.45	2.34
40	11.5	5	0.45	3.63
50	16.3	5	0.45	5.45
60	22.6	5	0.45	8.18

**Table 2:** Fit parameters for Equation (5) for the subplots shown in Figure 8.

$T\;\left[{}^{\circ }C\right]$	$B_{G^{\prime }}^{*}\;\left[mT\right]$	$B_{G^{\prime \prime }}^{*}\;\left[mT\right]$	$K_{G^{\prime }}^{*}\;\left[-\right]$	$K_{G^{\prime \prime }}^{*}\;\left[-\right]$
20	60 ±4	34 ±7	1 ,11 ±0 ,02	1 ,08 ±0 ,04
40	73 ±13	61 ±10	1 ,18 ±0 ,08	1 ,18 ±0 ,07

**Table 3:** Slopes for a linear scaling of ${\mathrm{MRE}}_{\mathrm{Ref}}$ with $\phi_{p}$ in Figure 9.

$T\;\left[{}^{\circ }C\right]$	$K_{G^{\prime }}\;\left[-\right]$	$K_{G^{\prime \prime }}\;\left[-\right]$
20%	16.8±1.8	18.1±1.6
40	0 1.5±0.2	0 1.7±0.2

**Table 4:** Fit parameters for Equation (10) for the data shown in Figure 10.

	$n\;\left[-\right]$	$C\;\left[-\right]$
${\mathrm{MRE}}_{G^{\prime }}$	−0.50±0.07	6.5±0.7
${\mathrm{MRE}}_{G^{\prime \prime }}$	−0.52±0.06	6.8±0.6

**Table 5:** Statistical significance for the approximation of $G^{\prime }$ and $G^{\prime \prime }$ with drift effects using a linear function with a slope $\frac{\Delta G}{\Delta t}$ and an intercept $G_{0}$.

	$p(\frac{\Delta G}{\Delta t})<0.01$	$p(\frac{\Delta G}{\Delta t})>0.01$	$p(G_{0})<0.01$	$p(G_{0})>0.01$
$G^{\prime }$	314	10	324	0
$G^{\prime \prime }$	296	28	324	0

**Table 6:** *p*-Values and $R^{2}$-values of the slopes for a linear scaling of ${\mathrm{MRE}}_{\mathrm{Ref}}$ with $\phi_{p}$ in Figure 9.

$T\;\left[{}^{\circ }C\right]$	$p\left(K_{G^{\prime }}\right)\;\left[-\right]$	$R^{2}\;\left[-\right]$	$p\left(K_{G^{\prime \prime }}\right)\;\left[-\right]$	$R^{2}\;\left[-\right]$
20	$5.8\times 10^{-8}$	0.979	$1.7\times 10^{-8}$	0.984
40	$9.4\times 10^{-8}$	0.976	$6.5\times 10^{-7}$	0.961

**Table 7:** *p*-Values of the slopes and intercepts and *R*^2^-values for a linear scaling of log $\frac{{\mathrm{MRE}}_{\mathrm{Ref}}}{\phi_{p}}$ with log $G^{\prime }$ in Figure 10, as described by Equation (10).

	$p\left(n\right)\;\left[-\right]$	$p\left(C\right)\;\left[-\right]$	$R^{2}\;\left[-\right]$
${\mathrm{MRE}}_{G^{\prime }}$	$7.5\times 10^{-11}$	$4.0\times 10^{-11}$	0.934
${\mathrm{MRE}}_{G^{\prime \prime }}$	$9.4\times 10^{-12}$	$3.8\times 10^{-13}$	0.949

## Data Availability

The data presented in this study are available on request from the corresponding author.

## Supplementary Materials

The following supporting information can be downloaded at: https://www.mdpi.com/article/10.3390/gels9030182/s1, Supplementary Figures S1–S4. Storage and loss modulus over time equivalent to Figure 4 for $w_{s}=10\%$ and $T=20\;{}^{\circ }C$, $w_{s}=10\%$ and $T=40\;{}^{\circ }C$, $w_{s}=60\%$ and $T=20\;{}^{\circ }C$ and $w_{s}=60\%$ and $T=40\;{}^{\circ }C$.

## Abbreviations

The following abbreviations are used in this manuscript:

LCST	Lower critical solution temperature
MRE	Magnetorheological effect
NIPAAm	*N*-isopropylacrylamide
