# Supplementary material for: Empirical Law for the Magnetorheological Effect of Nanocomposite Hydrogels with Magnetite Microparticles
Source: Gels. 2023 Feb 25;9(3):182. doi: 10.3390/gels9030182 (PMC10048087; doi:10.3390/gels9030182)
Supplement: Supplementary file 1 [file gels-09-00182-s001.zip › gels-2185725-supplementary.pdf]

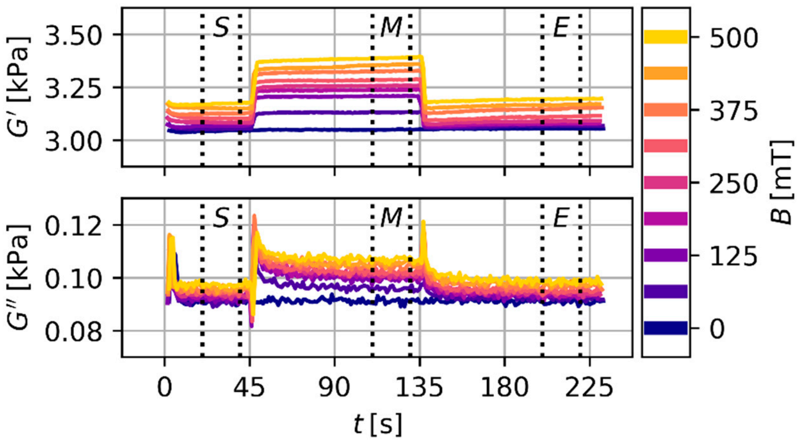

Supplementary Figure S1: Storage and loss modulus over time. The magnetic field is activated at t = 45 s and deactivated at t = 135 s. Measurements are conducted successively with increasing magnetic flux density for a single sample. w_s *= 10 % and T = 20 °C*

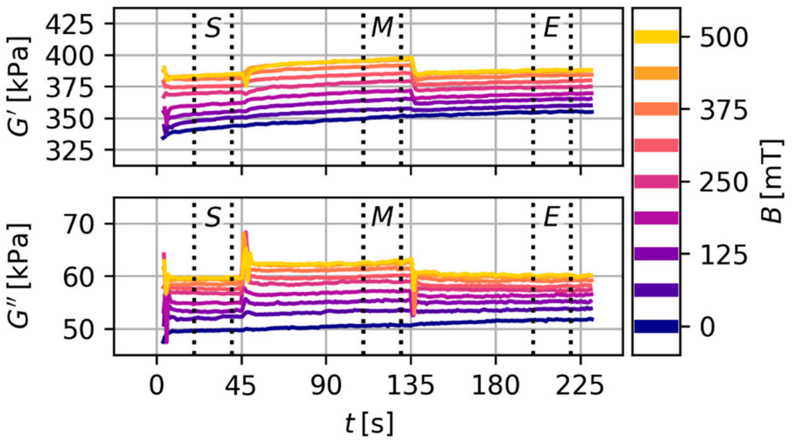

Supplementary Figure S2: Storage and loss modulus over time. The magnetic field is activated at t = 45 s and deactivated at t = 135 s. Measurements are conducted successively with increasing magnetic flux density for a single sample. w_s *= 10 % and T = 40 °C*

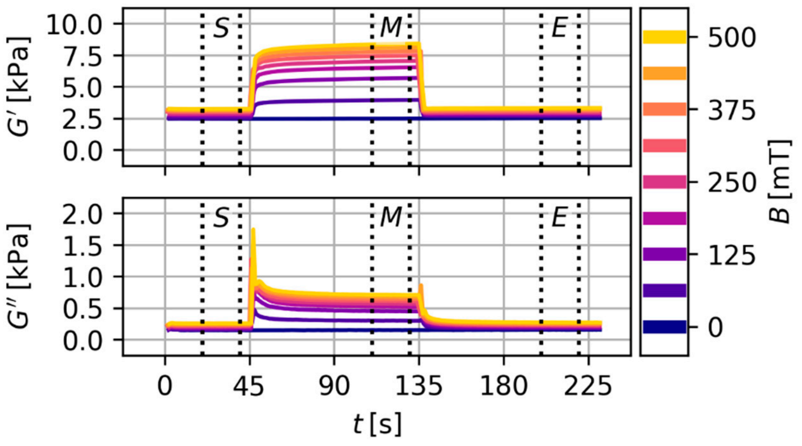

Supplementary Figure S3: Storage and loss modulus over time. The magnetic field is activated at t = 45 s and deactivated at t = 135 s. Measurements are conducted successively with increasing magnetic flux density for a single sample. w_s *= 60 % and T = 20 °C*

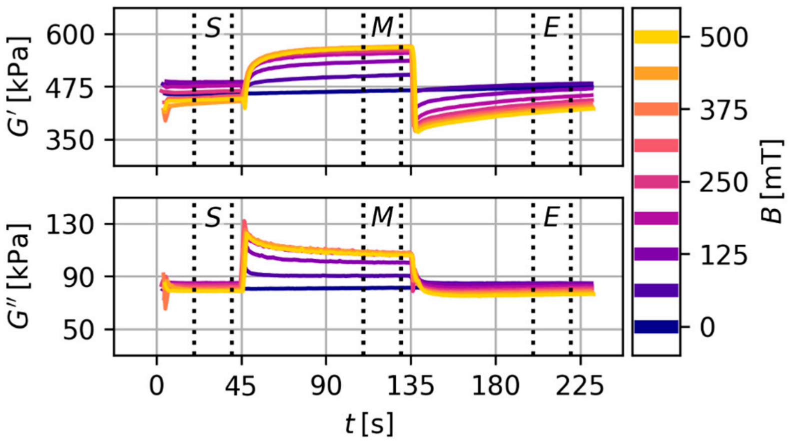

Supplementary Figure S4: Storage and loss modulus over time. The magnetic field is activated at t = 45 s and deactivated at t = 135 s. Measurements are conducted successively with increasing magnetic flux density for a single sample. w_s *= 60 % and T = 40 °C. Only this combination of* w_s *and T shows a decrease of G' after the deactivation of the magnetic field. The reason for this different behavior is yet unknown.*
